# Toward Uncovering the Complexities of Bacterial Interspecies Communication and Competition on the Skin

**DOI:** 10.1128/mbio.01320-22

**Published:** 2022-07-25

**Authors:** Francis Alonzo

**Affiliations:** a Department of Microbiology and Immunology, College of Medicine, University of Illinois Chicago, Chicago, Illinois, USA

**Keywords:** quorum sensing, *Staphylococcus hominis*, skin infection, Agr system, *Staphylococcus aureus*, skin microbiota

## Abstract

The skin is an inhospitable environment for microbial growth and survival. Hallmarks of the skin microenvironment include low moisture, high acidity, high lipid content, and paucity of essential nutrients, which together establish an antimicrobial barrier that defends against pathogens. Yet, commensal microbes and some opportunistic pathogens call this harsh environment home. The coagulase-negative staphylococci (CoNS) comprise a major constituent of the commensal skin microbiome. Of the CoNS, Staphylococcus epidermidis and Staphylococcus hominis are two common colonizers of human skin. Although comparatively less studied than S. epidermidis, there is a growing appreciation for S. hominis as a beneficial commensal, prompting interest in understanding the mechanisms by which S. hominis interacts with other skin microbes, including those with pathogenic potential. In their recent work, M. M. Severn, M. R. Williams, A. Shahbandi, Z. L. Bunch, et al. [mBio 13(3):e00930-22, 2022, https://doi.org/10.1128/mbio.00930-22] explore quorum sensing as a mediator of S. hominis interbacterial communication that can reduce the virulence of pathogens.

## COMMENTARY

Interspecies interactions among commensal microbiota are instrumental to tissue and organ homeostasis, prevention of disease, and resistance to colonization by pathogenic microorganisms (1). The protective capacity of commensal bacteria is driven by diverse adaptive traits that also promote survival of the bacterium itself in inhospitable host environments. The microenvironment of healthy skin is an excellent example of the synergy that exists between host-driven innate defenses against infection, commensal adaptation to these innate defenses, and commensal-driven exclusion of pathogenic microbes (2). Breakdown or dysfunction in any of these facets has the potential to stimulate changes in the composition of the microbiota that can lead to uncontrolled inflammatory conditions and infections of the skin such as atopic dermatitis (AD). Understanding the interplay between commensal and pathogenic microbes within the skin microenvironment can shed new light on how interspecies competition for a niche directs tissue homeostasis or the development of disease.

The coagulase-negative staphylococci (CoNS) are a main constituent of the commensal microbiota of the skin, with Staphylococcus epidermidis being the most frequently isolated species (3). S. epidermidis promotes skin barrier integrity and facilitates infection resistance via the release of antibacterial factors; however, it is also a cause of opportunistic infection—often associated with implants and medical devices (4). These divergent commensal and pathogenic traits highlight the complex relationship between presumably beneficial microbes and the host and emphasize the importance of their continued study. Like S. epidermidis, Staphylococcus hominis is a commensal bacterium commonly found on the skin of healthy individuals (3). Most notorious as one of the primary agents of human body odor, S. hominis is routinely isolated from the axillae and other skin sites commonly associated with malodor (5). Nevertheless, several studies also implicate S. hominis in skin protection and colonization resistance toward opportunistic pathogens such as Staphylococcus aureus via the release of lantibiotics (6).

In addition, two recent studies highlight the relevance of S. hominis peptide-based communication systems in the competitive establishment of its niche on the skin (7, 8). Like other staphylococcal species, S. hominis harbors an accessory gene regulatory (Agr) system that controls gene expression via a secreted cyclic peptide known as an autoinducing peptide (AIP). The cyclic peptide is processed and released by the bacterial cell and activates its cognate two-component system (AgrC/AgrA) to induce gene expression. The Agr system is well characterized in S. aureus and has been studied in several other Gram-positive pathogens, including S. epidermidis, Listeria monocytogenes, and Clostridioides difficile. The Agr system of S. aureus controls the expression of the genes that promote virulence. In 2019, Gless et al. identified a cyclic AIP (AIP-III) from S. hominis that had inhibitory activity against the Agr system of S. aureus (8). Around the same time, Williams et al. identified an S. hominis cyclic AIP variant (AIP-I) that not only inhibited S. aureus Agr signaling when purified AIP-I was applied to the skin but also prevented disease when S. aureus was coadministered with S. hominis in a murine model of AD (7). These studies suggested that peptide-based interference with Agr signaling could serve as a mode of niche competition for S. hominis that also has the potential to drive a healthy skin microenvironment by diminishing the pathogenic propensity of opportunistic pathogens.

**FIG 1:**
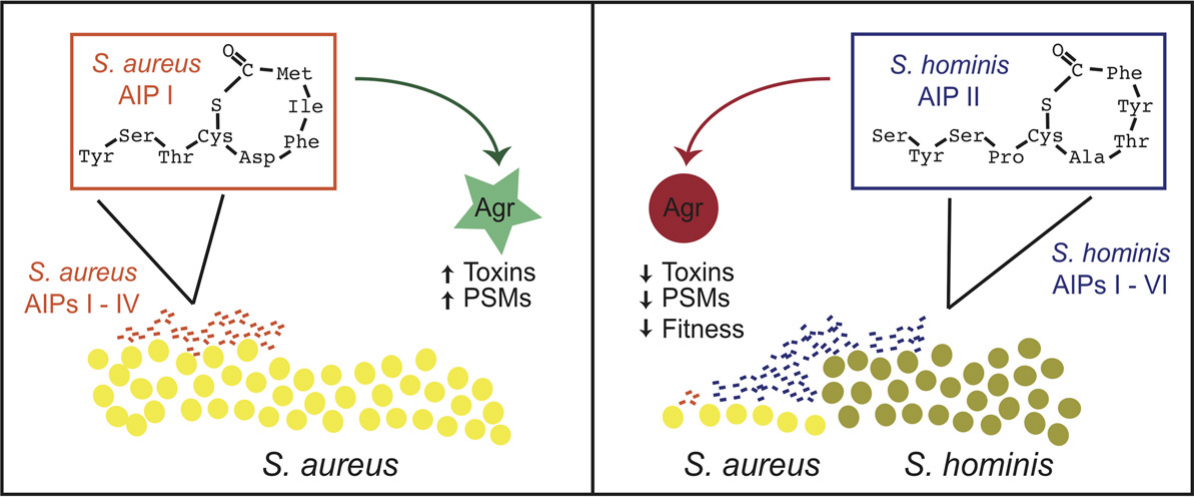
S. hominis promotes a healthy skin environment through interspecies inhibition of quorum sensing. (Left) S. aureus produces AIPs (I to IV) to induce the production of virulence factors when at high cell density. (Right) The commensal bacterium S. hominis produces at least six novel AIPs which can inhibit Agr systems of other species, including S. aureus, leading to reductions in virulence factor gene expression and pathogenesis. PSMs, phenol-soluble modulins.

In their recent *mBio* study, Severn et al. provided new insight into the complexities underlying interspecies quorum sensing inhibition through systematic identification of cyclic AIP variants in S. hominis isolates and an assessment of how these AIP variants impact quorum sensing in S. aureus and S. epidermidis (9) (Fig. 1). Using a combination of sequence analyses from genomes of S. hominis isolates and liquid chromatography-mass spectrometry (LC-MS)-based approaches to identify novel S. hominis AIPs from these same strains, the authors identified at least six unique AIP variants. The idea that a single bacterial species has strains that encode different AIP variants is not uncommon. S. aureus strains can encode one of four AIP variants, whereas some CoNS can encode three (Staphylococcus simulans) to four (S. epidermidis). However, S. hominis appears to have the most diverse repertoire of AIP variants of any CoNS studied to date. Thus, this new work highlights the potential for tremendous diversity in cyclic autoinducing peptides from Gram-positive bacteria and highlights the wealth of potential impacts they could have on cocolonizing bacteria in the skin.

One of the major strengths of this study is its comprehensive and systematic characterization of the impacts of each newly identified S. hominis AIP on Agr system activation in both S. aureus and S. epidermidis. Using an Agr-regulated transcriptional reporter, the authors determined that some S. hominis AIPs are potent inhibitors of Agr system activation (of several S. aureus and S. epidermidis Agr variants), some have no inhibitory potential, and others may even activate certain Agr systems. Furthermore, S. hominis AIPs appear to also undergo intraspecies competition. S. hominis AIP-I and AIP-II potently inhibit the activity of their noncognate AgrC/AgrA two-component systems. These observations, coupled with the determination that Agr-I- and Agr-II-containing S. hominis strains were enriched on the skin of a small cohort of healthy individuals, implicate quorum sensing inhibition in the establishment of the overall makeup of the healthy skin microbiota. The various inhibitory and activating potentials of S. hominis AIP variants among species and within a single species suggest a network of signaling events that, at minimum, could drive niche competition and exclusion of pathogenic microbes.

The extensive *in vitro* studies prompted a logical next step by the authors, which was to determine the extent to which one of the newly identified S. hominis AIP variants (AIP-II) protects against damage caused by the infectious bacterium S. aureus. In models of dermonecrosis and epicutaneous infection, the application of synthetic AIP-II abrogated skin damage caused by S. aureus. These data make a compelling argument for the notion that quorum sensing inhibition could conceivably drive reductions in skin pathology associated with S. aureus infection. Furthermore, coadministration of S. hominis encoding AIP-II and S. aureus to the skin led to a trend toward significant reductions in skin pathology, arguing that the cocolonized state could dictate the severity of infection outcome. These studies open up areas for broader exploration into the roles of quorum sensing and interspecies competition in colonization and how they contribute to the progression of disease and/or maintenance of healthy skin.

Overall, this compelling work underscores the importance of considering the constituents of the microbiome in the establishment and maintenance of healthy skin. Furthermore, it supports the prevailing view that bacterial cell-cell communication systems can have a tremendous impact on neighboring microbes and can even mitigate disease or exclude pathogenic microbes by driving a transcriptional profile that leads to a fitness disadvantage. This study provides a comprehensive assessment of how interspecies interactions might impact skin health through characterization of cyclic peptides produced by S. hominis. While S. hominis isolates from the skin were evaluated in this study, the observations have the potential to be applicable to a wide range of microbes that produce quorum-sensing signals in various tissue sites. Some areas for immediate and future investigation include (i) broader sequence analysis to determine the extent of Agr variants in skin-colonizing S. hominis, (ii) a more comprehensive profiling of healthy volunteers to determine the extent of S. hominis colonization relative to Agr type and assessment of any correlates with disease incidence, (iii) direct evaluation of how strains with different Agr types might shape other constituents of the skin microbiome, and (iv) exploration of the genes regulated by the S. hominis Agr system and how they might also contribute to commensalism or establishment of healthy human skin.
